# Effectiveness of Noise Cancelling Earbuds in Reducing Hearing and Auditory Attention Deficits in Children with Autism

**DOI:** 10.3390/jcm13164786

**Published:** 2024-08-14

**Authors:** Julien Zanin, Dani Tomlin, Gary Rance

**Affiliations:** Department of Audiology and Speech Pathology, The University of Melbourne, 550 Swanston St Carlton, Melbourne, VIC 3053, Australia; julien.zanin@unimelb.edu.au (J.Z.); dtomlin@unimelb.edu.au (D.T.)

**Keywords:** earbud technologies, assistive listening devices, autism spectrum disorder, auditory attention, speech perception

## Abstract

**Background/Objectives**: Autism spectrum disorder (ASD) is a lifelong neurodevelopmental condition characterised by impairments in social communication, sensory abnormalities, and attentional deficits. Children with ASD often face significant challenges with speech perception and auditory attention, particularly in noisy environments. This study aimed to assess the effectiveness of noise cancelling Bluetooth earbuds (Nuheara IQbuds Boost) in improving speech perception and auditory attention in children with ASD. **Methods:** Thirteen children aged 6–13 years diagnosed with ASD participated. Pure tone audiometry confirmed normal hearing levels. Speech perception in noise was measured using the Consonant-Nucleus–Consonant-Word test, and auditory/visual attention was evaluated via the Integrated Visual and Auditory Continuous Performance Task. Participants completed these assessments both with and without the IQbuds in situ. A two-week device trial evaluated classroom listening and communication improvements using the Listening Inventory for Education-Revised (teacher version) questionnaire. **Results:** Speech perception in noise was significantly poorer for the ASD group compared to typically developing peers and did not change with the IQbuds. Auditory attention, however, significantly improved when the children were using the earbuds. Additionally, classroom listening and communication improved significantly after the two-week device trial. **Conclusions:** While the noise cancelling earbuds did not enhance speech perception in noise for children with ASD, they significantly improved auditory attention and classroom listening behaviours. These findings suggest that Bluetooth earbuds could be a viable alternative to remote microphone systems for enhancing auditory attention in children with ASD, offering benefits in classroom settings and potentially minimising the stigma associated with traditional assistive listening devices.

## 1. Background

Autism spectrum disorder (ASD), commonly referred to as “autism”, is a heritable lifelong neurodevelopmental condition with diverse cognitive features. There is considerable variation in reported prevalence, but recent estimates suggest that (worldwide) approximately 1 in 100 children are affected [1].

Key manifestations of ASD include impairments in social communication and interaction, delayed development of non-verbal and verbal communication skills, repetitive behaviours, and varying degrees of intellectual disability [2]. Additionally, sensory abnormalities represent a pervasive aspect of ASD, affecting around 90% of individuals with the disorder [3]. These abnormalities include a range of atypical sensory experiences, manifesting as heightened or diminished responsiveness to stimuli across the auditory, visual, tactile, gustatory, and olfactory modalities.

Hearing impairment is a relatively common manifestation of ASD and was reported in Kanner’s original paper describing the disorder [4]. Peripheral hearing loss is more prevalent than in the general population, with 2–5% suffering impaired sound detection thresholds [5,6,7]. Furthermore, >50% of affected individuals present with auditory processing deficits affecting the ability to hear and understand speech [8,9,10,11,12,13,14]. These central abnormalities include disruption of temporal processing (the ability to perceive subtle timing variations in auditory signals), binaural integration (the capacity to combine auditory signals presented to the left and right ears), and spatial stream segregation (the use of interaural difference cues to localise target sound sources within a noisy listening environment). The functional consequence of these basic processing deficits is impaired perception of complex acoustic signals (such as speech) in everyday listening situations [12,13,15].

Speech perception difficulties are further exacerbated by attentional deficits. Studies by Kemner et al. (1995) [16] and Ceponiene et al. (2003) [17] report a reduced capacity to attend to and process linguistic sounds in individuals with autism. Furthermore, children with ASD have been shown to have less finely-tuned auditory spatial attention, meaning that they are more likely to be distracted by extraneous sounds and have greater difficulty filtering irrelevant information when attending to acoustic targets [18,19]. This is especially important in school-aged children with autism, as the ability to identify and attend to speech in a noisy classroom has been suggested to be the most significant predictor of academic development [20].

Remote microphone technology has shown promise in reducing some of the functional hearing deficits experienced by children with ASD [13,21,22,23]. This type of technology consists of an ear-level receiver worn by the child, which is wirelessly connected to a microphone (worn at the lapel) by the child’s teacher or communication partner. By recording the speaker’s voice in close proximity to the mouth, the transmitted signal is relatively loud compared to the level of the background noise when perceived by the listener. There are, however, some limitations with these devices, including cost, stigma, and reluctance to use the device by the teacher and/or child [24,25]. Additionally, as only one person can wear the transmitter at any time, the benefit of remote microphone technology is limited in situations where there is more than one primary speaker—such as family mealtimes and student-led group work [26].

One potential alternative to remote microphone systems is Bluetooth wireless earbuds. These are direct-to-consumer devices, some of which offer amplification of the acoustic signal, automatic noise reduction, and directional microphone technology [27,28]. In general, there has been limited research on the therapeutic efficacy of earbud devices, especially for children with ASD; however, there is some evidence to suggest that the noise reduction and speech enhancement features can improve speech perception for individuals with normal hearing acuity but with self-reported hearing difficulties [27,29]. Moreover, these devices may overcome some of the inherent limitations of remote microphone systems previously discussed.

The aim of this study was to determine whether over-the-counter Bluetooth earbuds equipped with noise cancelling and directional microphone technology (Nuheara IQbuds Boost) can improve speech perception and auditory attention in the presence of background noise for children diagnosed with ASD.

## 2. Methods

### 2.1. Participants

The study involved thirteen children (10 males) aged between 6 and 13 years with a mean ± SD age of 9.2 ± 2.2 years. Each participant had been diagnosed with ASD via a multidisciplinary clinical assessment using a range of instruments, including the Autism Diagnostic Interview and the Childhood Autism Rating Scale. None of the children had known coexisting disabilities, and all had normal cognition (Full-Scale IQ [Wechsler Intelligence Scale for Children] values > 70). Each child used oral communication, was able to follow verbal instruction and attended their local mainstream school where they had a consistent classroom teacher.

### 2.2. Pure Tone Audiometry

Sound detection thresholds were assessed via an Interacoustics AD226 diagnostic audiometer in a quiet room where noise levels were confirmed using a sound level meter to be <40 dB sound pressure level (SPL). Participant hearing levels were measured at octave frequencies between 500 and 8000 Hz and were deemed normal if thresholds were ≤20 dB hearing level [30].

### 2.3. Noise Cancelling Earbuds

Nuheara IQbuds (Boost) are wireless earbuds that enable the streaming of audio signals via Bluetooth-compatible devices. They are coupled to the wearer’s ear canal using silicone or foam ear tips, which come in a range of different sizes to ensure a tight and secure fit. The adequacy of the fitting can be assessed using the Ear ID feature available on a mobile phone application (IQbuds). In addition to Bluetooth streaming capabilities, the IQbuds are equipped with a directional microphone array and adaptive noise cancellation which are designed to improve the clarity of the signal provided to the listener. The directional microphone array works by leveraging the physical and acoustic design features of the device (including the placement and distance between the three microphones on each earbud) to preferentially capture sound from the front while attenuating noise from other directions. In particular, directional microphone arrays use variations in the timing of sound reaching the different microphones to generate a phase difference between sounds coming from different directions. These phase differences cause sounds from unwanted directions (side and rear) to cancel each other out, improving the salience of the target signal. Adaptive noise cancellation is another technique by which background noise may be minimised through the use of multiple microphones. In this case, both external and in-ear microphones acquire background noise components in the sound environment. These microphone signals are then filtered and added to the earbud hearing path signal. The multiple acoustic signals combine and cancel each other out, reducing the level of background noise presented to the listener.

For the Nuheara IQbuds, these features can be activated by the wearer through a mobile phone application, giving them access to sounds in their environment. Both the directional microphone array and adaptive noise cancelling can potentially improve the wearer’s ability to hear speech in the presence of competing background noise [31].

### 2.4. Speech Perception in Background Noise

Discrimination of open-set speech perception ability was evaluated using the Consonant-Nucleus–Consonant-Word test [32]. The assessment was carried out in the free field with the participant seated between two loudspeakers and facing the front. A speaker situated 2 m in front presented a series of single-syllable words calibrated to reach the child at 65 dB SPL, while a rear speaker provided background noise (recorded 4-talker babble) at the same level. As such, the signal-to-noise ratio (SNR) was 0 dB at the child’s head. This ratio was selected to simulate the listening conditions in a typical school classroom [33]. The presentation distance (2 m) was selected to reflect a typical speaking distance for a teacher addressing a student in the centre of the classroom. The participant was required to imitate the stimulus words, and a percentage of correctly discriminated speech sounds (phonemes) was calculated by the examiner. Testing was completed with and without the Nuheara IQbuds *in-situ* and the test order (earbud-aided first versus unaided first) was randomised for each participant. The IQbuds were set up using the Ear ID feature and fit to each participant by an audiologist (JZ) to ensure the correct earbud size was selected.

Speech perception results obtained from the ASD participant group were subsequently compared to a set of control data (*n* = 20) obtained from a group of normally hearing/developing children of similar age published by Rance et al. (2014) [13]. The participants from this study were evaluated using the same speech test material and an identical test setup.

### 2.5. Attention in Background Noise Assessment

Auditory and visual attention was assessed using the integrated visual and auditory continuous performance task–quick screen (IVA-QS; [34]), which has been shown to be sensitive to improvements in attention in children using remote microphone technologies [35]. The IVA-QS test was administered on a Hewlett Packard Probook laptop connected to a Behringer Monitor MS16 loudspeaker. This loudspeaker was positioned 2 m in front of the participant and played the IVA-QS auditory stimuli (i.e., target stimulus). Another MS16 loudspeaker was placed 2 m behind the participant and played 4-talker babble concurrently. Both signals were calibrated to reach the participant at 65 dB SPL (0 dB SNR). Participants were instructed to click a computer mouse (placed in the dominant hand) whenever they heard the number “one” or saw it on the laptop screen. They were also instructed to ignore all number “two” stimuli (which were also presented auditorily and visually) and were randomly interspersed throughout the test. The duration of the IVA-QS assessment was 8 min, and participants were tasked with completing the test with- and without the IQbuds in situ (total test time of 16 min). The order in which the testing was administered was randomised, and testing in the two conditions was carried out on separate days, approximately 2 weeks apart, to optimise participant cooperation and minimise the effects of testing fatigue.

The IVA-QS assessment provides metrics on auditory, visual, sustained auditory, and sustained visual attention. Auditory and visual attention scores are computed from measures of vigilance, focus, and speed. Vigilance is indicated by two distinct types of omission errors during the test: speed represents the average response time for correct responses, and focus measures the overall variability of mental processing speed for correct responses. The sustained attention subscale for each modality offers a comprehensive measure of the ability to respond to stimuli consistently and accurately under low-demand conditions while also sustaining attention and adapting under high-demand conditions when the stimuli undergo changes. Results on the IVA-QS are normalised such that a typically developing child would score 100, and a clinically abnormal child would score ≤ 70.

### 2.6. Device Trial

The children were fit binaurally with IQbuds and were instructed to wear the devices as much as possible over a 2-week trial period. A two week trial duration has been shown to be sufficient to demonstrate auditory benefit in studies involving participants with ASD [13] and in other paediatric populations [36].

Usage in noisy environments (at school and at home) was encouraged. Device benefits were assessed by each child’s primary classroom teacher before and after the 2-week device trial.

### 2.7. Listening Inventory for Education-Revised (LIFE-R)

The effect of the IQbuds on classroom listening and communication was assessed using the Teacher version of the Listening Inventory for Education-Revised (LIFE-R) questionnaire [37]. This survey requires that the teacher estimate the challenges experienced by the student in a set of 15 commonly occurring classroom-based scenarios. Estimates of listening and communication competence are recorded using a 5-point Likert scale, where a score of 5 indicated “no challenge”, and a score of 1 suggested that the participant was “always challenged”.

## 3. Results

### 3.1. Pure Tone Audiometry and Loudness Discomfort Levels

Hearing thresholds were within the normal range (≤20 dBHL) for all 13 participants at each of the test frequencies (250 Hz–8000 Hz). As such, no amplification was provided through the earbuds for the perceptual and attention assessments or the 2-week device trial. Four-frequency average (500/1000/2000/4000 Hz) hearing levels for each individual are shown in Table 1.

### 3.2. Speech Perception in Background Noise

Discrimination of speech in background noise was abnormal. A two-sample *t*-test indicated that the group of autistic children in the present cohort had significantly poorer binaural speech perception in noise than reported for normally developing children of a similar age (*t*(14) = 10.58, *p* < 0.001) [13]. Furthermore, each of the 12 participants (who were able to complete the testing) showed clinically abnormal monosyllabic perception. Phoneme identification scores for each individual are shown in Table 1.

**Figure 1:**
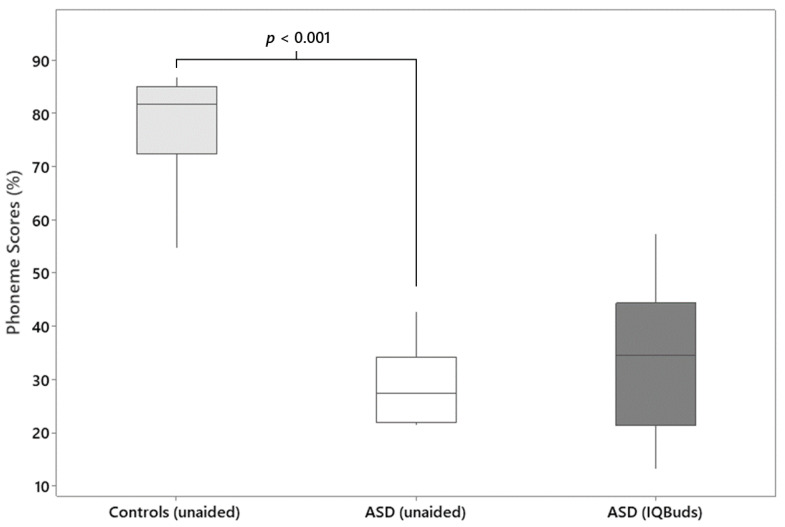
Boxplots showing the percentage of phonemes correctly identified in background noise (0 dB SNR). Findings are for a group of control participants (*n* = 20) published by Rance et al. (2014) [13] and for children with confirmed autism spectrum disorder (ASD) (*n* = 13) in 2 conditions: unaided and aided (with IQBuds in situ). The boxes represent the median, 25th and 75th percentiles. Minimum and maximum values are depicted by the end of the whiskers.

Provision of the IQBuds did not significantly improve speech perception in background noise. The mean earbud-aided phoneme score was 33.5 ± 13.3%, which was not significantly higher than that obtained for the unaided condition (27.0 ± 15.7%) (*t* = 1.4, *p* = 0.19). A summary of the group speech perception results is shown in Figure 1.

### 3.3. Attention in Background Noise

Auditory attention was impaired for the ASD participants in this study. Three of the 12 children able to complete the IVO-QS task showed clinically abnormal results (<70% standardised score), and a fourth was unable to respond reliably to auditory stimuli despite being able to complete the visual component of the task (Table 1). A chi-squared goodness-of-fit test found this proportion of abnormal results (4/12) to be significantly higher than expected for a normal population (*p* < 0.001). In contrast, visual attention was typically normal, with all but one of the participants (who were able to complete the task) showing either visual attention or visual sustained attention scores within the normal range (*p* > 0.05) (Table 1).

**Figure 2:**
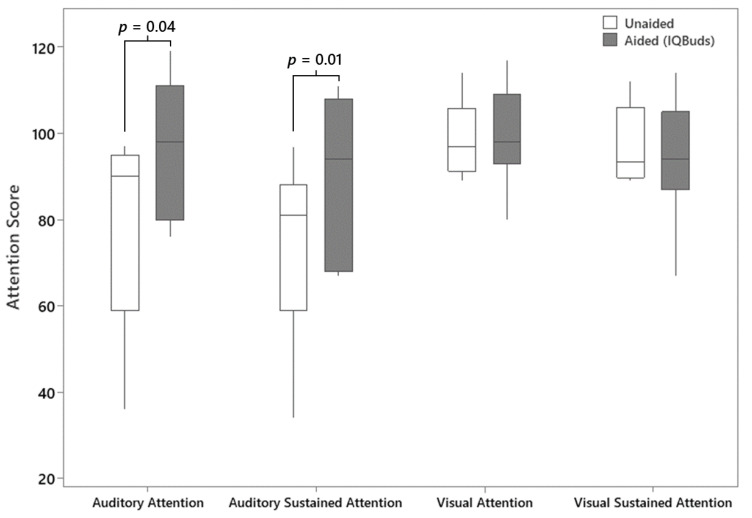
Boxplots showing attention scores obtained by the participants with ASD on each domain of the Integrated Visual and Auditory Continuous Performance Task: auditory attention, auditory sustained attention, visual attention, and visual sustained attention. Results showed a statistically significant improvement in aided (IQBuds in situ) condition compared to unaided for both auditory attention assessments. The boxes represent the median, 25th and 75th percentiles. Minimum and maximum values are depicted by the end of the whiskers.

The provision of the IQbuds significantly improved auditory attention in background noise. A two-sample paired *t*-test revealed an improvement in the aided (IQBuds) condition compared to the unaided condition for both measures (auditory attention: *t* = 2.64, *p* = 0.04; auditory sustained attention: *t* = 3.77, *p* = 0.01) (Figure 2). Additionally, Cohen’s d effect size calculations revealed a large effect for both the auditory attention (d = 0.92) and auditory sustained attention measures (d = 0.81) when participants were using the devices. In contrast, averaged results for the visual attention and visual sustained attention measures showed no change when participants were tested using the earbuds (*p* > 0.05) (Figure 2).

### 3.4. Device Trial

Of the thirteen study participants, only ten were able to complete the two-week earbud trial. One child (ASD10) rejected the devices at the initial fitting session as he was unable to tolerate the earpieces due to tactile hypersensitivity. The other two participants (ASD4 and ASD5) stopped wearing the devices in the latter stages of the trial as the earbuds were too big to sit comfortably (and remain securely) in their ears. As a result, findings for experiments involving pre- and post-trial comparisons involved 10 children. At the conclusion of the trial, eight of the ten opted to continue using the device.

### 3.5. Listening and Communication Disability

Classroom listening, communication and participation were considered to have improved significantly with the provision of the earbud devices. The mean pre-trial (unaided) Teacher LIFE-R percentage score was 44.4 ± 16.0%, whereas the post-trial (aided) score was 55.3 ± 23.3%. This represented a significant improvement in classroom listening for the participants over the two-week trial period (*t* = −3.05, *p* = 0.02).

## 4. Discussion

This study sought to establish whether wireless Bluetooth earbuds (Nuheara IQbuds) could improve listening behaviours in background noise for children with ASD. We found that while speech perception ability was unaffected by the directional- and noise cancellation processing provided by the devices, they did afford significant improvements in auditory attention and school classroom listening.

The speech perception findings were in agreement with the literature [13,15,39]. Results showed that despite having normal hearing acuity, the ASD cohort had significantly more difficulty understanding speech in the presence of background noise than expected for normally developing children of equivalent age. Each child, in fact, presented with perceptual deficits severe enough to exacerbate the communication challenges central to autism spectrum disorder [13]. Interestingly, the processed signal provided by the earbud devices did not significantly improve perceptual ability in background noise. This result is somewhat surprising and may be a reflection of the laboratory test setup. The placement of the loudspeaker 2 m from the participant may have affected the ability of the device microphone array to adequately enhance the target signal, as directional technologies are most effective at shorter distances [40]. This is due to an increasing reduction in the intensity of the sound source over distance as well as an increase in the negative effect of reverberation. Furthermore, while noise cancellation technology has been shown to improve selective attention [41], listening comfort [42], and reduce listening effort [43], there is a paucity of evidence indicating noise cancelling algorithms can enhance speech intelligibility in the presence of background noise [44]. Most important, however, was the fact that perceptual ability was not impaired by either the occlusive presence of the earbud in the auditory canal or the signal processing provided by the devices.

Auditory attention was highly variable and clinically abnormal in 30% of the study participants. This result is consistent with previously reported findings for children with ASD [10,45] and may be attributable to difficulties in using spatial cues to segregate speech from competing noise. Spatial listening is directly linked to attention and involves a top-down hierarchical prediction at the auditory cortex, which is compared to the incoming signal. Any discrepancy between the signal and the prediction is transmitted back up to the level of the auditory cortex. Poor attention mechanisms reduce the auditory cortex’s precision in this bidirectional signal matching, leading to poorer speech perception in noisy environments [35,46]. Studies further support that attention-priming mechanisms can compensate for degraded signals caused by central auditory nervous system pathologies, thereby improving spatial listening abilities [47,48].

Importantly, our study revealed significant improvements in attention with the provision of earbud devices. Analysis of the group data showed increases in both auditory attention and auditory sustained attention in background noise. The auditory attention score is generated in equal parts by measures of vigilance (omission errors), focus (variability in processing speed for correct responses), and speed (average reaction time for correct responses) [49]. Comparatively, auditory sustained attention is a global measure of the ability to respond accurately, quickly, and reliably under low-demand conditions while also assessing the capacity to sustain attention and be flexible under high-demand conditions. The sustained attention score is generated based on acuity and reliability, elasticity and steadiness, and dependability and swiftness [49]. Overall, auditory attention deficits may adversely impact reading fluency [50], which has been shown to strongly correlate with reading, writing, spelling, grammar and numeracy skills [51,52,53]. In addition to an overall improvement in auditory attention and auditory sustained attention scores with the participants wearing the earbuds, two of the four clinically abnormal participants improved to within the normal auditory attention range when device-aided. As such, the findings suggest a similar degree of benefit to that reported for remote-microphone devices in school-aged children with listening-in-noise deficits [35].

Earbud use also translated into improved classroom behaviours in the classroom trial participants. Prior to the 2-week trial, teacher estimates of classroom listening, communication and participation on the LIFE-R questionnaire showed a mean score of 44%, suggesting that on average, the children “sometimes or often” experienced challenges in the classroom. There are no published norms for the Teacher-LIFE-R inventory, but this rating level is poorer than reported for children with peripheral hearing loss who require hearing aids or cochlear implants [54].

Classroom behaviour scores improved significantly with the provision of earbud devices. This benefit was not universal (Table 1), but overall, the teacher-LIFE-R ratings increased to the point where the average student was “only occasionally” experiencing classroom difficulties. Importantly, eight of the ten children who took part in the 2-week trial opted to continue using the earbuds at the study’s conclusion. This high device uptake may have reflected their improved ability to concentrate in acoustically challenging learning environments. In addition, it may have reflected a greater degree of listening comfort when using the devices. A high proportion of children with ASD are known to suffer hyperacusis (where sounds of low or moderate intensity are perceived as excessively loud [55] and the noise reduction provided by the devices may have made sounds more tolerable in the busy classroom.

### 4.1. Clinical Implications

In general, the availability of evidence-based options designed to improve auditory attention and reduce the impact of auditory processing deficits for children is limited. Currently, remote microphone technology is the most frequently recommended assistive technology, and the benefits are well supported by the research literature [35,56,57]. There are, however, some limitations with the use of remote microphone systems that could potentially hinder or restrict its use for certain individuals. For instance, the technology is costly (approximately 5–6 times that of standard earbuds) and, depending on the country, is typically only covered by public health funding for children with significant hearing loss (i.e., impaired sound detection ability) [26,58]. Additionally, several studies have identified other barriers to the uptake and consistent use of remote microphone technology. These include stigma associated with wearing the ear-level receiver (which is typically designed to look like a hearing aid), resistance from teachers and lack of benefit in situations with multiple speakers [25,26,59].

Comparatively, Bluetooth earbuds present a promising alternative due to their direct-to-consumer availability, affordability, relatively discreet design and ubiquity (in non-therapeutic contexts), which may reduce the stigma associated with their use. Furthermore, Bluetooth earbuds are more versatile as they do not rely on a (second) device to transmit the speaker’s voice and can, therefore, be used in non-structured settings, including during group activities in the classroom and social contexts.

Three children failed to complete the device trial. One child, who suffered severe tactile hypersensitivity, could not tolerate the earpiece for more than a few moments and failed to progress beyond the initial fitting stage. Similar issues have been reported previously with ear-level auditory devices but do appear to be relatively rare, at least in high functioning ASD populations [13]. The other two children were able to wear the earbuds but found them uncomfortable—reporting that the earpieces were too big to fit comfortably within the bowl of the concha. Future device designs will need to account for developmental changes in pinna anatomy [60,61] to optimise comfort and useability in pre-teen populations.

### 4.2. Study Limitations

The study had several limitations. Firstly, the small sample size may limit the generalisability of the study findings to the broader population and may reduce statistical power. Despite this, the study still revealed a large effect size between the unaided and earbud-aided attention scores. Secondly, the duration of the device trial in this study was relatively short, spanning a period of only 2 weeks. Other studies trialling auditory devices in autistic children of equivalent age have shown significant improvements within this timeframe [13,62,63], but it is possible that this limited period may not have captured the full extent of the potential benefits (or limitations) of the earbud devices. Longitudinal studies with longer trial and follow-up periods are necessary to assess the sustained effects of the intervention and evaluate its long-term efficacy and usability in real-world settings. Finally, the study did not blind the researchers to the device condition during speech perception testing, which could potentially bias the results. Similarly, the schoolteachers were aware that their students were wearing the device during the trial period. Future studies may consider blinding the teacher to device status, but previous studies attempting this (with the student wearing the auditory system through “on” and “off” phases) have proven challenging [63].

## 5. Conclusions

The findings from this study further support the need for comprehensive audiological assessment in the diagnosis and management of autism, emphasising how important it is to address the effects of auditory processing deficits in educational settings. Furthermore, the study elucidates the potential of using direct-to-consumer wireless Bluetooth earbuds as a novel intervention to address the auditory attention deficits commonly experienced by children with ASD. Future research should aim to explore the long-term efficacy and usability of these devices in real-world settings, with the aim of better understanding their potential role in improving the daily lives of children with ASD.

## Figures and Tables

**Table 1:** Participant demographics and individual test results.

Participant	Sex	Age	4FA (L)	4FA (R)	CNC Unaided	CNC Aided	Auditory Attention Unaided	Auditory Attention Aided	Sustained Auditory Attention Unaided	Sustained Auditory Attention Aided	Visual Attention Unaided	Visual Attention Aided	Sustained Visual Attention Unaided	Sustained Visual Attention Aided	LIFE-R Unaided	LIFE-R Aided
ASD1	M	10	10	11.25	42.7	57.3	59	98	59	68	92	98	94	94	*	*
ASD2	M	11	11.25	3.75	28.0	33.0	92	119	87	111	99	100	90	105	33.3	50.7
ASD3	M	7	13.75	11.25	1.3	13.3	95	108	81	104	*	*	*	*	41.0	41.0
ASD4	F	9	7.5	5	60.0	46.7	97	111	97	108	114	117	112	114	*	*
ASD5	F	7	3.75	7.5	2.7	49.3	*	*	*	*	*	109	*	102	29.3	29.3
ASD6	M	9	11.25	13.75	36.0	20.0	79	92	80	94	89	94	89	94	*	*
ASD7	M	8	17.5	16.25	24.0	37.3	90	76	88	87	103	93	104	87	74.7	96.0
ASD8	F	13	6.25	8.75	21.3	17.3	36	80	34	67	95	80	93	67	56.0	78.0
ASD9	M	7	12.5	11.25	26.7	25.3	54	*	44	*	85	*	89	*	*	*
ASD10	M	6	7.5	5	DNT	DNT	DNT	DNT	DNT	DNT	DNT	DNT	DNT	DNT	DNT	DNT
ASD11	M	9	8.75	8.75	25.0	36.0	99	104	74	105	100	102	92	108	*	*
ASD12	M	12	8.75	8.75	28.0	30.0	103	*	104	98	113	118	108	90	43.0	49.0
ASD13	M	11	10	12.5	28.4	36.7	*	*	*	*	20	27	0	34	33.3	43.0
Mean (Std Dev)		9.2 (2.2)	9.9 (3.5)	9.5 (3.7)	27.0 (15.7)	33.5 (13.3)	80.4 (22.8)	98.5 (15.1)	74.8 (22.6)	93.6 (16.5)	91.0 (26.7)	93.8 (26.1)	87.1 (31.7)	89.5 (23.5)	44.4 (16.0)	55.3 (23.3)

Age: age at assessment (years); 4FA: 4-frequency average hearing level (dBHL); CNC: Consonant-nucleus-consonant word test–phoneme score (%); LIFE-R: Listening Inventory for Education-Revised (%); ATTENTION: IVO-QS (Standardised scores); results in red represent scores outside published normative ranges (CNC word scores < 68% [13]; IVO-QS Scores < 70 [38]). The asterisks indicate data points that were not completed.

## Data Availability

The data presented in this study are available on request from the corresponding author due to ethical reasons.
